# Serum Methylarginines and Spirometry-Measured Lung Function in Older Adults

**DOI:** 10.1371/journal.pone.0058390

**Published:** 2013-05-15

**Authors:** Mark A. McEvoy, Peter W. Schofield, Wayne T. Smith, Kingsley Agho, Arduino A. Mangoni, Roy L. Soiza, Roseanne Peel, Stephen J. Hancock, Ciriaco Carru, Angelo Zinellu, John R. Attia

**Affiliations:** 1 Centre for Clinical Epidemiology & Biostatistics, Hunter Medical Research Institute, School of Medicine & Public Health, University of Newcastle, Newcastle, New South Wales, Australia; 2 Centre for Translational Neuroscience and Mental Health, University of Newcastle, Newcastle, New South Wales, Australia; 3 Environmental Health Branch, New South Wales Department of Health, Gladesville, New South Wales, Australia; 4 Science of Mental Health & Adversity Unit, School of Medicine, University of Western Sydney, Parramatta, New South Wales, Australia; 5 Division of Applied Medicine, School of Medicine and Dentistry, University of Aberdeen, Aberdeen, United Kingdom; 6 Department of Medicine for the Elderly, Woodend Hospital, NHS Grampian, Aberdeen, United Kingdom; 7 Department of Biomedical Sciences, University of Sassari, Sassari, Italy; 8 Department of General Medicine, John Hunter Hospital, Newcastle, New South Wales, Australia; University College London, United Kingdom

## Abstract

**Rationale:**

Methylarginines are endogenous nitric oxide synthase inhibitors that have been implicated in animal models of lung disease but have not previously been examined for their association with spirometric measures of lung function in humans.

**Objectives:**

This study measured serum concentrations of asymmetric and symmetric dimethylarginine in a representative sample of older community-dwelling adults and determined their association with spirometric lung function measures.

**Methods:**

Data on clinical, lifestyle, and demographic characteristics, methylated arginines, and L-arginine (measured using LC-MS/MS) were collected from a population-based sample of older Australian adults from the Hunter Community Study. The five key lung function measures included as outcomes were Forced Expiratory Volume in 1 second, Forced Vital Capacity, Forced Expiratory Volume in 1 second to Forced Vital Capacity ratio, Percent Predicted Forced Expiratory Volume in 1 second, and Percent Predicted Forced Vital Capacity.

**Measurements and Main Results:**

In adjusted analyses there were statistically significant independent associations between a) higher asymmetric dimethylarginine, lower Forced Expiratory Volume in 1 second and lower Forced Vital Capacity; and b) lower L-arginine/asymmetric dimethylarginine ratio, lower Forced Expiratory Volume in 1 second, lower Percent Predicted Forced Expiratory Volume in 1 second and lower Percent Predicted Forced Vital Capacity. By contrast, no significant associations were observed between symmetric dimethylarginine and lung function.

**Conclusions:**

After adjusting for clinical, demographic, biochemical, and pharmacological confounders, higher serum asymmetric dimethylarginine was independently associated with a reduction in key measures of lung function. Further research is needed to determine if methylarginines predict the decline in lung function.

## Introduction

Lung health and obstructive lung disease (OLD) are considered health priorities in Australia and worldwide due to the significant burden of disease associated with a decline in lung function. Age-related decline in lung function is associated with increased risk of OLD [1] as well as cardiovascular [2] and all-cause mortality [3]. Age-related decline in lung function is not well understood, however smoking and asthma are well established risk factors [4], [5]. Lung function may also decline in the absence of *established* risk factors and a search for *additional* risk factors is clearly warranted.

Nitric oxide (NO), a key endogenous mediator, is well known for its effects on cardiovascular function but less well known for its role in lung physiology and pathophysiology. In the lung, NO plays a major role in airway and vascular smooth muscle relaxation, ventilation perfusion matching, neurotransmission, mucociliary clearance, airway mucus secretion, and host defence [6]. It is also involved in the pathophysiology of lung diseases including pulmonary hypertension, airway hyper-responsiveness (AHR), asthma, chronic obstructive pulmonary disease (COPD), and Cystic Fibrosis (CF) [6], [7].

In alveolar endothelial cells, airway epithelial cells, and endothelial cells of the bronchial and pulmonary circulation, NO is synthesised from the amino acid L-arginine by the constitutive enzyme isoform endothelial nitric oxide synthase (eNOS) [8]. In airway cholinergic nerves, airway epithelial cells, and type 1 pneumocytes, NO is also synthesised by the constitutive enzyme isoform neuronal nitric oxide synthase (nNOS) [9]. An inducible nitric oxide synthase isoform (iNOS) is also expressed in epithelial and inflammatory cells of the airway following exposure to proinflammatory cytokines, producing large amounts of NO relative to that produced by eNOS and nNOS [10]. Altered expression of each NOS isoform has been documented in a number of lung diseases [11].

Given the importance of NO in lung physiology and its role in airway pathology, a disturbed synthesis and/or availability of NO might contribute to the development of impaired lung function and airway disease. The methylarginine asymmetric dimethylarginine (ADMA) is an endogenous inhibitor of NOS [12] that inhibits the activity of all three NO synthase isoforms, and has been shown to reduce NO concentrations in numerous cell types [12], [13], [14], [15], [16]. Elevated plasma ADMA concentrations have been consistently demonstrated in individuals with traditional vascular risk factors and those with existing cardiovascular disease [17]. Symmetric dimethylarginine (SDMA) is a related molecule that has been reported to competitively inhibit arginine uptake in vitro [17], potentially implicating it in vascular disease and other conditions dependent on adequate NO availability [18].

Endogenous methylarginines are important potentially modifiable molecules that may be associated with impaired or declining lung function and airway disease. This view is supported by the following observations.

1. ADMA and SDMA limit NO availability through NOS inhibition and/or L-arginine availability and NO is required for normal lung function [6].
2. NO is responsible for the maintenance of normal pulmonary endothelial function and recent studies have shown that pulmonary endothelial dysfunction is associated with asthma and COPD [19], all conditions in which NO synthesis and/or availability is impaired.
3. It has recently been demonstrated that the lung is not only a major source of NO but is also a major source of the NOS inhibitor ADMA [20]. Bulau et al. demonstrated in mice that pulmonary expression of type I protein arginine methyltransferase 1 was correlated with enhanced protein arginine methylation and pulmonary ADMA degradation was undertaken by the isoform 1 of the enzyme dimethylarginine dimethylaminohydrolase (DDAH1). Furthermore, bronchoalveolar lavage fluid and serum exhibited almost identical ADMA/SDMA ratios. Together, these observations suggest that methylarginine metabolism by the pulmonary system significantly contributes to circulating ADMA and SDMA concentrations.
4. In both chronic pulmonary hypertensive rats and patients with idiopathic pulmonary arterial hypertension there is a marked increase in serum and tissue ADMA and SDMA concentrations [21].
5. In mice, ADMA affects physiology and collagen formation in the airways through alteration in the L-arginine metabolizing pathways [22]. Consistent with this finding, dysregulation in L-arginine metabolism is associated with airflow abnormalities in severe asthma patients [23].
6. Pharmacological inhibitors of NOS have been used to study the role of specific NOS isoforms in asthma and have provided support for iNOS-mediated NO production in the potentiation of airway inflammation [24]. In contrast, overexpression of eNOS attenuates airway inflammation in a murine model of allergic asthma [25]. It has been demonstrated that in a mouse model of allergic asthma, increased lung ADMA concentrations and decreased DDAH expression are associated with airway inflammation following allergen challenge [26]. These findings provide support for the role of endogenous ADMA in allergen-induced lung inflammation.
7. ADMA concentrations are also increased in human asthma lung and sputum samples [27].

Hence an increase in endogenous ADMA and/or SDMA, may contribute to the underlying pathology of decline in lung function in humans through a direct influence on NOS isoforms and/or depletion of L-arginine which is required for adequate synthesis of NO. Given that no previous study has examined the association of endogenous methylarginines with spirometric lung function, the primary aim of this research is to measure serum concentrations of the methylarginines ADMA and SDMA in a representative sample of older community-dwelling adults and determine their association with spirometric measures of lung function. We show that in a sample of older community-dwelling adults, increased serum ADMA is associated with reduced measures of lung function as measured by spirometry.

## Methods

Data for this study was obtained from the Hunter Community Study (HCS), a cohort of community-dwelling men and women aged 55 to 85 years of age in Newcastle, New South Wales (NSW), Australia. Approval to conduct the research was granted by the University of Newcastle Human Research Ethics Committees and written informed consent was obtained from all study participants. This study has been described in detail elsewhere [28]. In brief, participants were randomly selected from the New South Wales State Electoral roll and 9,784 individuals were contacted between December 2004 and May 2007. Of these, a total of 3,253 actually participated (response rate 44.5% after removing incorrect addresses and non-contacts). Participants completed a series of self-reported questionnaires, attended a clinic visit, and consented to linkage of health records. For full details of this cohort and sample characteristics see McEvoy M. et al., 2010 [28]. The sample for this investigation (n = 500) was derived from the initial cohort by simple random sampling. Of the 500 subjects randomly selected there were complete exposure and outcome data for 483 subjects.

### Main exposure variables: L-arginine and methylarginines

The primary explanatory variables were serum ADMA and SDMA concentrations. Secondary variables were L-arginine, and L-arginine/ADMA ratio. As L-arginine is the substrate for NOS-dependent synthesis of NO we hypothesize that higher L-arginine, and L-arginine/ADMA ratio will be associated with improved lung function. Blood was collected in EDTA tubes and centrifuged at 4°C and 3000 g for 10 min. to separate serum, which was stored for three years at −80°C before analysis. L-Arginine and its di-methylated forms (ADMA and SDMA) were measured in serum by hydrophilic- interaction liquid chromatography and isotope dilution tandem mass spectrometry [29]. The intra and inter-assay CV's for arginine, ADMA and SDMA were all less than 15%.

#### Primary outcomes

Spirometry was used to measure Forced Expiratory Volume in 1 sec. (FEV1) and Forced Vital Capacity (FVC) and was performed using electronic spirometers (Micro Medical SpiroUSB, Cardinal Health, Kent, UK) with Spida 5 software (Carefusion Ltd, Kent, UK) and predicted values of Gore [30]. The five key lung function measures included as outcomes in this investigation were FEV1, FVC FEV1/FVC, %Predicted FEV1, and %Predicted FVC. Spirometers were calibrated daily using a 3-L syringe. All values obtained were corrected to body temperature and ambient pressure, saturated with water vapour (BTPS), with an assumed fixed room temperature and atmospheric pressure. Lung function measures were measured in accordance with the American Thoracic Society (ATC) guidelines [31].

### Study factors

A number of factors were chosen *a priori* for testing association with lung function. These factors were selected based on existing knowledge derived from reviewing the literature and included the participant's age, gender, marital status, education level, household income, height, smoking status, alcohol intake, self-reported medical & surgical history (clinician diagnosis), mean systolic blood pressure, mean diastolic blood pressure, pulse pressure, number of general practitioner visits, Short Form-36 physical function score (Pf), Centre for Epidemiologic Studies Depression Scale depressive symptom score (CESD depressive symptoms), self-reported medication use, step count, Body Mass Index (BMI), Waist-to-Hip Ratio (WHR), creatinine, urea, urate, Glomerular Filtration Rate (GFR), fasting glucose, HDL cholesterol (HDL), LDL cholesterol (LDL), triglycerides, white blood cell count (WBC), haemoglobin, serum fibrinogen, serum homocysteine (measured using laser-induced fluorescence capillary electrophoresis) and serum C-reactive protein (CRP) (measured using latex-enhanced immunoturbidimetry).

### Statistical Analysis

The statistical analysis was performed using STATA software version 12.0 (Stata Corporation, College Station, TX, USA). The analyses include twenty models that examine the association of the L-arginine and methylarginine measures (ADMA, SDMA, L-arginine, and L-arginine/ADMA ratio) with each of the five lung function measures. Multiple linear regression was used to examine the associations, initially with simple linear regression analysis for each factor and then followed by multivariate analyses, to assess the independent effects after controlling for other potential confounders or covariates. The models were constructed by backward elimination using the following procedures: 1) only variables with p-value<0.20 in the univariate analysis were entered into the models for backward elimination; 2) the screened variables (potential confounders) were included in the model and the non-significant variables (p>0.05) were manually eliminated step by step and 3) any variables removed from the final regression model due to collinearity were reported. The main predictor variable (ADMA, SDMA, L-Arginine or L-Arginine/ADMA) was retained in all the final models. The coefficients with 95% confidence intervals were calculated in order to assess the adjusted risk of independent variables, and those with p<0.05 were retained in the final model.

## Results

From the sample of 483 participants [median age (IQR) = 64 (60–70) years; Females = 54%], the prevalence of obstructive ventilatory defect defined by the Global Initiative for Chronic Obstructive Lung Disease (GOLD) as a FEV_1_/FVC ratio below 70% was 6.6% (N = 32). The means of each lung function outcome (FEV1, FVC, FEV1/FVC, %Predicted FEV1, and %Predicted FVC) were 2.48 litres (S.D = 0.69), 2.99 litres (S.D = 0.83), 0.83 (S.D. = 0.08), 89.95% (S.D = 16.46), and 86.02% (S.D = 14.91) respectively. Each predictor (ADMA, SDMA, L-arginine, and L-arginine/ADMA ratio) was divided into quartiles of serum values in mmol/L with quartile 1 (Q1) as the lowest level (reference level) and quartile 4 (Q4) the highest level. The median plasma concentrations of each primary predictor within the lowest and highest quartile are described in Table 1. Participant characteristics in the lowest and highest quartile of each primary predictor are described in Table 2.

**Table 1:** Median serum concentration of Asymmetric dimethylarginine (ADMA), Symmetric dimethylarginine (SDMA), L-arginine, and L-arginine/ADMA ratio in lowest (Quartile 1) and highest (Quartile 4) quartile of each predictor (N = 483).

Predictor	Quartile 1 (95% CI)*	Quartile 4 (95% CI)
**ADMA (µmol/L)**	0.45 (0.45–0.46)	0.64 (0.63–0.65)
**SDMA (µmol/L)**	0.54 (0.53–0.55)	0.96 (0.94–0.99)
**L-arginine (µmol/L)**	32.7 (31.4–34.0)	80.2 (78.2–82.1)
**L-arginine/ADMA ratio**	61.3 (58.7–64.0)	148.3 (144.2–152.3)

* 95% confidence interval.

**Table 2:** Participant characteristics and mean of each lung function outcome in the lowest vs. highest quartile of ADMA, SDMA, L-arginine, and L-arginine/ADMA ratio in the Hunter Community Study.

		Methylarginine							
Characteristic	Sub group/or mean (S.D)	Asymmetric dimethylarginine (ADMA)		Symmetric dimethylarginine (SDMA)		L-arginine		L-arginine/Asymmetric dimethylarginine (L-arginine/ADMA ratio)	
		Quartile 1	Quartile 4	Quartile1	Quartile 4	Quartile 1	Quartile 4	Quartile 1	Quartile 4
**Age**	**mean (S.D)**	63.0 (0.5)	67.9 (0.8)	63.1 (0.5)	69.8 (0.8)	65.0 (0.6)	65.9 (0.7)	66.2 (0.7)	64.6 (0.7)
**Gender**	**Male (%)**	41.3	50.8	44.6	40.0	50.4	43.3	52.9	36.7
	**Female (%)**	58.7	49.2	55.4	60.0	49.6	56.7	47.1	63.3
**Body Mass Index**	**mean (S.D)**	28.0 (0.4)	29.1 (0.5)	30.0 (0.4)	27.7 (0.4)	29.5 (0.4)	28.8 (0.4)	29.4 (0.5)	28.5 (0.4)
**Smoking status**	**Never**	55.4	51.7	49.6	51.7	50.8	47.5	49.1	47.5
	**Ever**	40.5	36.7	47.1	45.0	46.7	47.5	47.5	45.8
	**Now**	4.1	11.6	3.3	3.3	2.5	5.0	3.4	6.7
**Highest level education**	**Primary or secondary school not completed**	15.7	24.3	19.6	27.9	23.0	20.5	24.8	17.5
	**Secondary school completed**	20.0	23.4	22.3	15.3	23.9	17.9	24.8	15.8
	**Trade or technical college qualification**	33.0	30.5	27.7	31.5	28.3	34.8	27.4	36.8
	**University or other tertiary qualification**	31.3	21.6	30.4	25.3	24.8	26.8	23.0	29.9
**Income**	**<A$40000/year (%)**	42.1	63.7	41.2	63.2	52.2	55.6	54.9	56.9
	**≥A$40000/year (%)**	57.9	36.3	58.8	36.8	47.8	44.4	45.1	43.1
**Asthma**	**Yes (%)**	8.5	19.2	10.9	16.7	11.6	16.1	12.4	13.7
	**No (%)**	91.5	80.8	89.1	83.3	88.4	83.9	87.6	86.3
**Atrial fibrillation**	**Yes (%)**	4.3	10.8	5.0	15.8	6.6	93.2	9.1	5.1
	**No (%)**	95.7	89.2	95.0	84.2	93.4	6.8	90.9	94.9
**Type 2 diabetes**	**Yes (%)**	11.1	10.0	16.0	9.2	11.6	9.3	11.6	11.1
	**No (%)**	88.9	90.0	84.0	90.8	88.4	90.7	88.4	88.9
**No. General practitioner visits**	**0–2 times**	20.3	16.8	23.1	13.6	14.2	26.7	15.8	29.2
	**3–6 times**	60.8	57.1	55.4	58.5	60.8	52.5	57.5	50.0
	**7+ times**	18.9	26.1	21.5	27.9	25.0	20.8	26.7	20.8
**LDL cholesterol**	**mean (S.D)**	3.3 (0.1)	3.0 (0.1)	3.2 (0.1)	3.0 (0.1)	3.0 (0.1)	3.2 (0.1)	3.0 (0.1)	3.3 (0.1)
**HDL cholesterol**	**mean (S.D)**	1.4 (0.03)	1.3 (0.03)	1.3 (0.03)	1.3 (0.03)	1.3 (0.03)	1.3 (0.04)	1.3 (0.03)	1.3 (0.04)
**Urate**	**mean (S.D)**	0.3 (0.01)	0.3 (0.01)	0.3 (0.01)	0.4 (0.01)	0.3 (0.01)	0.3 (0.01)	0.3 (0.01)	0.3 (0.01)
**C-reactive protein**	**mean (S.D)**	3.4 (0.5)	3.4 (0.3)	3.4 (0.5)	3.1 (0..3)	3.9 (0.5)	3.0 (0.3)	4.0 (0.5)	3.0 (0.3)
**Homocysteine**	**mean (S.D)**	9.0 (0.2)	10.5 (0.3)	9.0 (0.2)	11.2 (0.4)	10.4 (0.3)	9.4 (0.3)	10.8 (0.3)	9.1 (0.3)
**Glomerular filtration rate (GFR)**	**mean (S.D)**	84.5 (1.3)	73.7 (1.5)	87.9 (1.3)	66.9 (1.3)	79.4 (1.6)	77.6 (1.5)	78.0 (1.6)	80.0 (1.5)
**FEV1** * **(L)**	**mean (S.D)**	2.73 (0.06)	2.32 (0.06)	2.61 (0.06)	2.43 (0.07)	2.46 (0.06)	2.58 (0.06)	2.36 (0.06)	2.69 (0.06)
**FVC** † **(L)**	**mean (S.D)**	3.27 (0.07)	2.87 (0.08)	3.12 (0.08)	2.93 (0.07)	2.94 (0.07)	3.08 (0.08)	2.83 (0.07)	3.22 (0.08)
**FEV1/FVC** ‡	**mean (S.D)**	0.84 (0.01)	0.81 (0.01)	0.84 (0.01)	0.83 (0.01)	0.84 (0.01)	0.84 (0.01)	0.84 (0.01)	0.84 (0.01)
**%Predicted FEV1** §	**mean (S.D)**	93.4 (1.3)	87.0 (1.7)	89.9 (1.3)	88.7 (1.6)	89.1 (1.5)	92.2 (1.6)	87.8 (1.5)	92.6 (1.5)
**%Predicted FVC** ∥	**mean (S.D)**	88.7 (1.3)	85.4 (1.5)	84.8 (1.2)	85.9 (1.4)	84.4 (1.4)	87.5 (1.5)	83.5 (1.4)	87.6 (1.4)

* Forced Expiratory Volume in 1 second,

† Forced Vital Capacity,

‡ Forced Expiratory Volume in 1 second to Forced Vital Capacity ratio,

§ Percent Predicted Forced Expiratory Volume in 1 second,

∥ Percent Predicted Forced Vital Capacity.

Simple linear regression analyses of quartiles of ADMA, SDMA, L-arginine, and L-arginine/ADMA ratio with FEV1, FVC, FEV1/FVC, %Predicted FEV1, and %Predicted FVC are described in **Table 3**. In unadjusted analyses higher ADMA was statistically significantly associated with a reduction in all lung function measures. Furthermore, there was a general trend toward a greater reduction with increasing concentrations of ADMA. With the exception of FEV1/FVC, higher L-arginine/ADMA ratio was statistically significantly associated with an increase in all lung function measures. In unadjusted analyses, SDMA was only statistically significantly associated with FEV1. There was no association between L-arginine and any of the lung function measures.

**Table 3:** Unadjusted β -coefficients and 95% confidence intervals obtained from simple linear regression analyses of Asymmetric dimethylarginine (ADMA), Symmetric dimethylarginine (SDMA), L-arginine, and L-arginine/asymmetric dimethylarginine with Forced Expiratory Volume in 1 second (FEV1), Forced Vital Capacity (FVC), Forced Expiratory Volume in 1 second to Forced Vital Capacity ratio (FEV1/FVC), Percent Predicted Forced Expiratory Volume in 1 second (%Predicted FEV1), and Percent Predicted Forced Vital Capacity (%Predicted FVC).

Spirometric lung function measure																
		FEV1			FVC			FEV1/FVC			%Predicted FEV1			%Predicted FVC		
Predictor	Quartile	β*	95% CI†	p‡	β	95% CI	p	β	95% CI	p	β	95% CI	p	β	95% CI	p
**ADMA**	Ref															
	2	**−0.254**	**−0.420, 0.087**	**0.003**	**−0.311**	**−0.514, −0.108**	**0.003**	0.004	−0.015, 0.023	0.666	−2.33	−6.09, 1.43	0.225	−2.79	−6.30, 0.71	0.118
	3	**−0.347**	**−0.516, −0.179**	**<0.001**	**−0.427**	**−0.625, −0.229**	**<0.001**	0.002	−0.018, 0.021	0.870	**−5.03**	**−8.94, −1.13**	**0.012**	**−4.73**	**−8.39, −1.07**	**0.011**
	4	**−0.413**	**−0.584, −0.241**	**<0.001**	**−0.407**	**−0.614, −0.199**	**<0.001**	**−0.025**	**−0.046, −0.004**	**0.020**	**−6.40**	**−10.58, −2.21**	**0.003**	−3.35	−7.26, 0.58	0.094
**SDMA**	Ref															
	2	−0.152	−0.322, 0.018	0.080	−0.173	−0.380, 0.034	0.101	−0.004	−0.024, 0.015	0.662	1.19	−2.53, 4.90	0.531	1.97	−1.39, 5.32	0.251
	3	**−0.175**	**−0.346, −0.004**	**0.045**	−0.171	−0.386, 0.044	0.119	−0.012	−0.031, 0.006	0.185	0.38	−3.84, 4.61	0.859	1.91	−1.97, 5.78	0.334
	4	**−0.181**	**−.356, −0.007**	**0.042**	−0.186	−.393, 0.021	0.078	−0.014	−0.034, 0.007	0.191	−1.18	−5.22, 2.87	0.568	1.08	−2.57, 4.73	0.561
**L-arginine**	Ref															
	2	−0.059	−.234, 0.115	0.506	−0.027	−0.238, 0.184	0.798	−0.013	−0.033, 0.007	0.205	−0.11	−4.11, 3.88	0.956	1.74	−1.97, 5.45	0.358
	3	0.016	−0.148, 0.181	0.845	0.066	−0.128, 0.260	0.504	−0.017	−0.038, 0.004	0.118	0.39	−3.83, 4.61	0.855	1.79	−1.85, 5.42	0.335
	4	0.117	−0.049, 0.283	0.165	0.138	−0.067, 0.343	0.186	0.001	−0.018, 0.020	0.888	3.05	−1.15, 7.24	0.154	3.12	−0.87, 7.11	0.125
**L-arginine/ADMA ratio**	Ref															
	2	0.01	−.162, 0.182	0.909	0.084	−0.122, 0.290	0.422	−0.020	−0.042, 0.003	0.085	0.33	−3.91, 4.57	0.878	2.47	−1.31, 6.26	0.200
	3	0.134	−0.027, 0.300	0.102	0.164	−0.031, 0.359	0.098	−0.001	−0.020, 0.018	0.894	3.55	−0.46 7.57	0.083	3.65	0.002, 7.29	0.050
	4	**0.332**	**0.168, 0.496**	**<0.001**	**0.388**	**0.188, 0.587**	**<0.001**	0.003	−0.016, 0.023	0.733	**4.76**	**0.53 8.99**	**0.028**	**4.12**	**0.25 8.00**	**0.037**

* β -coefficient;

† 95% confidence interval;

‡ p-value.

In further analyses, age, gender, marital status, education, household income, height, asthma, angina, previous heart attack, high cholesterol, atrial fibrillation, hypertension, CESD depressive symptoms, alcohol, smoking years, number of general practice visits, Pf, urea, urate, LDL, HDL, fibrinogen, homocysteine, GFR, CRP, pulse pressure, and medications (consumption of cardiovascular, anticholinergic, NSAID medicines) were all statistically significantly associated with **FEV1** in a simple linear regression model. These same variables, with the exception of heart attack and use of anticholinergic medicines, were also statistically significantly associated with **FVC**. Furthermore, gender, marital status, education, household income, height, asthma, heart attack, atrial fibrillation, CABG, alcohol, number of general practice visits, urea, urate, and medications (consumption of cardiovascular and anticholinergic medicines) were statistically significantly associated with **FEV1/FVC** in a simple linear regression model.

We also explored the association of other potential predictors with %Predicted FEV1 and %Predicted FVC. Gender, marital status, education, household income, asthma, heart attack, high cholesterol, atrial fibrillation, hypertension, alcohol, smoking years, number of general practice visits, pf, urea, urate, WBCC, fasting glucose, fibrinogen, homocysteine, CRP, pulse pressure, and medications (consumption of cardiovascular and anticholinergic medicines) were all statistically significantly associated with %Predicted FEV1in univariate analysis. These same variables, with the exception of CABG and CESD depressive symptoms, were also statistically significantly associated with %Predicted FVC.

Multiple linear regression analyses of quartiles of ADMA, SDMA, L-arginine, and L-arginine/ADMA ratio with **FEV1** are described in **Table 4**. In adjusted analysis ADMA (Q3), gender, education, asthma, number of general practice visits, age, height, smoking years, Pf, urate, and fibrinogen remained statistically significant predictors of FEV1. In combination these variables explained 66.12% of the total variation in FEV1. Quartile 3 of ADMA concentration was associated with a reduction of FEV1 of 0.112 litres compared with Q1 while Q4 of ADMA concentration was associated with a reduction of FEV1 of 0.098 litres compared with Q1, but this did not reach statistical significance. In adjusted analysis L-arginine/ADMA ratio (Q4), gender, education, household income, asthma, Pf, and fibrinogen remained statistically significant predictors of FEV1. In combination these variables explained 62.71% of the total variation in FEV1. Quartile 4 of the L-arginine/ADMA ratio was associated with an increase of FEV1 of 0.130 litres compared with Q1. The analogous multivariate regression with %predicted FEV1 is shown in Table S1; the pattern of significant variables was similar as with FEV1 but was less marked. There were no statistically significant associations between SDMA and L-arginine with FEV1, however quartile 4 of L-arginine was associated with an increase in %Predicted FEV1 of approximately 3.7%.

**Table 4:** Adjusted β -coefficient and 95% confidence intervals obtained from a multiple linear regression analysis of Asymmetric dimethylarginine, symmetric dimethylarginine, L-arginine, and L-arginine/asymmetric dimethylarginine ratio with FEV1.

Characteristic	Adjusted			Adjusted			Adjusted			Adjusted		
	β*	95% CI†	*p* ‡	*β*	95% CI	*p*	*β*	95% CI	*p*	*β*	95% CI	*p*
Predictors	Asymmetric Dimethylarginine			Symmetric Dimethylarginine			L-Arginine			L-arginine/ADMA ratio		
Quartile 1	Ref			Ref			Ref			Ref		
Quartile 2	−0.036	−0.143, 0.072	0.513	0.062	−0.048, 0.172	0.268	−0.001	−0.103, 0.102	0.991	−0.022	−0.148, 0.105	0.736
Quartile 3	−0.112	−0.219, −0.004	**0.042**	−0.023	−0.145, 0.099	0.711	−0.012	−0.130, 0.106	0.839	0.047	−0.062, 0.155	0.401
Quartile 4	−0.098	−0.217, 0.022	0.108	0.065	−0.053, 0.183	0.278	0.099	−0.006, 0.204	0.064	0.130	0.015, 0.245	**0.027**
**Gender**												
Female	Ref			Ref			Ref			Ref		
Male	0.542	0.405, 0.678	<0.001	0.525	0.392, 0.658	<0.001	0.498	0.375, 0.622	<0.001	0.479	0.347, 0.610	<0.001
**Level of education**												
Primary or secondary not completed	Ref			Ref						Ref		
Completed secondary	0.093	−0.021, 0.207	0.109	0.100	−0.023, 0.224	0.111				0.162	0.034, 0.290	0.013
TAFE	0.078	−0.039, 0.194	0.193	0.095	−0.031, 0.221	0.139				0.135	0.008, 0.262	0.038
University or other tertiary study	0.124	0.012, 0.236	0.030	0.126	0.001, 0.250	0.047				0.176	0.046, 0.305	0.008
**Household income**												
<A$40,000				Ref			Ref			Ref		
> = A$40,000				0.098	0.001, 0.195	0.049	0.111	0.025, 0.196	0.011	0.122	0.021, 0.222	0.018
**Asthma**												
No	Ref			Ref			Ref					
Yes	−0.250	−0.413, −0.087	0.003	−0.288	−0.450, −0.126	0.001	−0.267	−0.417, −0.116	0.001	−0.268	−0.428, −0.107	0.001
**No. General practitioner visits**												
0–2 times	Ref											
3–6 times	0.000	−0.100, 0.099	0.996									
7+ times	−0.183	−0.326, −0.039	0.013									
**Age**	−0.027	−0.033, −0.021	<0.001	−0.028	−0.035, −0.022	<0.001	−0.028	−0.033, −0.022	<0.001	−0.027	−0.033, −0.021	<0.001
**Height**	0.023	0.015, 0.031	<0.001	0.022	0.014, 0.030	<0.001	0.023	0.015, 0.031	<0.001	0.021	0.013, 0.030	<0.001
**Smoking years**	−0.008	−0.010, −0.005	<0.001	−0.008	−0.011, −0.005	<0.001	−0.008	−0.011, −0.006	<0.001			
**Physical function score Pf**	0.003	0.001, 0.005	0.007	0.003	0.001, 0.005	0.001	0.003	0.001, 0.005	0.008	0.003	0.001, 0.006	0.005
**Urate**	−0.573	−1.134, −0.012	0.045									
**Fibrinogen**	−0.120	−0.195, −0.046	0.002	−0.142	−0.221, −0.064	<0.001	−0.131	−0.203, −0.059	<0.001	−0.163	−0.242, −0.085	<0.001
**Adjusted R-squared %**	**66.12**			**64.76**			**65.13**			**62.71**		

Independent variables adjusted for based on p-value<0.20 are: CESD depressive symptom score, gender, marital status, education, household income, height, asthma, angina, heart attack, high cholesterol, atrial fibrillation, hypertension, drink days per month, general practice visits, physical function score, urea, urate, LDL cholesterol, HDL cholesterol, fibrinogen, homocysteine, glomerular filtration rate, pulse pressure, C-reactive protein, cardiovascular medication use, anticholinergic medication use, non-steroidal anti-inflammatory drug use.

* β -coefficient;

† 95% confidence interval;

‡ p-value.

Multiple linear regression analyses of quartiles of ADMA, SDMA, L-arginine, and L-arginine/ADMA ratio with **FVC** are described in **Table 5**. In adjusted analysis ADMA (Q3), gender, education, age, height, smoking years, Pf, and fibrinogen remained statistically significant predictors of FVC. In combination these variables explained 66.40% of the total variation in FVC. Quartile 3 of ADMA concentration was associated with a reduction of FVC of 0.137 litres compared with Q1. The analogous multivariate regression with %predicted FVC is shown in Table S2; the pattern of significant variables was similar as with FVC but was less marked.

**Table 5:** Adjusted β -coefficient and 95% Confidence intervals obtained from a multiple linear regression analysis of Asymmetric dimethylarginine, symmetric dimethylarginine, L-arginine, and L-arginine/asymmetric dimethylarginine ratio with FVC.

Characteristic	Adjusted			Adjusted			Adjusted			Adjusted		
	β*	95% CI†	*p* ‡	*β*	95% CI	*p*	*β*	95% CI	*p*	*β*	95% CI	*p*
Predictors	Asymmetric Dimethylarginine			Symmetric Dimethylarginine			L-Arginine			L-arginine/ADMA ratio		
Quartile 1	Ref			Ref			Ref			Ref		
Quartile 2	−0.064	−0.193, 0.065	0.329	0.069	−0.057, 0.195	0.281	0.058	−0.069, 0.186	0.370	0.029	−0.102, 0.161	0.660
Quartile 3	−0.137	−0.264, −0.009	**0.035**	0.014	−0.130, 0.157	0.851	0.034	−0.094, 0.163	0.598	0.077	−0.049, 0.203	0.228
Quartile 4	−0.067	−0.211, 0.077	0.362	0.059	−0.073, 0.190	0.381	0.091	−0.042, 0.224	0.180	0.122	−0.009, 0.253	0.068
**Gender**												
Female	Ref			Ref			Ref			Ref		
Male	0.562	0.414, 0.710	<0.001	0.563	0.415, 0.711	<0.001	0.562	0.414, 0.709	<0.001	0.556	0.408, 0.703	<0.001
**Highest level of education**												
Primary or secondary not completed	Ref			Ref			Ref			Ref		
Completed secondary	0.137	0.001, 0.273	0.048	0.142	0.004, 0.281	0.044	0.144	0.005, 0.283	0.042	0.143	0.005, 0.280	0.043
TAFE	0.105	−0.036, 0.247	0.144	0.114	−0.028, 0.255	0.114	0.111	−0.029, 0.250	0.121	0.105	−0.035, 0.244	0.141
University or other tertiary study	0.184	0.045, 0.322	0.009	0.190	0.049, 0.331	0.008	0.189	0.047, 0.331	0.009	0.183	0.043, 0.322	0.011
**Age**	−0.033	−0.039, −0.026	<0.001	−0.034	−0.041, −0.028	<0.001	−0.034	−0.040, −0.028	<0.001	−0.033	−0.040, −0.027	<0.001
**Height**	0.034	0.025, 0.043	<0.001	0.035	0.025, 0.044	<0.001	0.034	0.025, 0.044	<0.001	0.034	0.025, 0.044	<0.001
**Smoking years**	−0.006	−0.009, −0.002	0.001	−0.006	−0.010, −0.003	<0.001	−0.006	−0.010, −0.003	<0.001	−0.006	−0.010, −0.003	<0.001
**Physical function score Pf**	0.004	0.002, 0.007	<0.001	0.004	0.002, 0.007	0.001	0.004	0.002, 0.007	0.001	0.004	0.002, 0.006	0.001
**Fibrinogen**	−0.190	−0.281, −0.099	<0.001	−0.200	−0.290, −0.110	<0.001	−0.197	−0.286, −0.109	<0.001	−0.197	−0.286, −0.109	<0.001
**Adjusted R-squared %**	**66.40**			**66.20**			**61.38**			**66.38**		

Independent variables adjusted for based on p-value<0.20 are: cesd depressive symptom score, gender, marital status, education, household income, height, asthma, angina, heart attack, high cholesterol, atrial fibrillation, hypertension, drink days per month, general practice visits, physical function score, urea, urate, LDL cholesterol, HDL cholesterol, fibrinogen, homocysteine, glomerular filtration rate, pulse pressure, C-reactive protein, cardiovascular medication use, anticholinergic medication use, non-steroidal anti-inflammatory drug use.

* β -coefficient;

† 95% confidence interval;

‡ p-value.

Multiple linear regression analyses of quartiles of ADMA, SDMA, L-arginine, and L-arginine/ADMA ratio with **FEV1/FVC** are described in **Table 6**. In adjusted analysis there was no statistically significant association with ADMA, SDMA, L-arginine, or L-arginine/ADMA ratio with FEV1/FVC. However, household income, asthma, smoking years, and height remained statistically significant predictors of FEV1/FVC in all four models.

**Table 6:** Adjusted β -coefficient and 95% confidence intervals obtained from a multiple linear regression analysis of Asymmetric dimethylarginine, symmetric dimethylarginine, L-arginine, and L-arginine/asymmetric dimethylarginine ratio with for the ratio FEV1/FVC.

Characteristic	Adjusted			Adjusted			Adjusted			Adjusted		
	β*	95% CI†	*p* ‡	*β*	95% CI	*p*	*β*	95% CI	*p*	*β*	95% CI	*p*
Predictors	Asymmetric dimethylarginine			Symmetric dimethylarginine			L-Arginine			L-arginine/ADMA ratio		
Quartile 1	Ref			Ref			Ref			Ref		
Quartile 2	0.006	−0.013, 0.024	0.550	−0.002	−0.019, 0.016	0.844	−0.014	−0.033, 0.005	0.152	−0.018	−0.040, 0.003	0.087
Quartile 3	0.005	−0.014, 0.024	0.592	−0.014	−0.032, 0.003	0.109	−0.015	−0.035, 0.006	0.164	−0.003	−0.021, 0.015	0.773
Quartile 4	−0.010	−0.030, 0.009	0.301	−0.003	−0.023, 0.016	0.739	0.009	−0.009, 0.027	0.320	0.010	−0.008, 0.028	0.264
**Household income**												
<A$40,000	Ref			Ref			Ref			Ref		
> = A$40,000	0.026	0.013, 0.039	<0.001	0.026	0.013, 0.039	<0.001	0.027	0.014, 0.040	<0.001	0.027	0.014, 0.040	<0.001
**Asthma**												
No	Ref			Ref			Ref			Ref		
Yes	−0.064	−0.091, −0.036	<0.001	−0.067	−0.094, −0.039	−0.068	−0.068	−0.095, −0.040	<0.001	−0.067	−0.094, −0.040	<0.001
**Smoking years**	−0.001	−0.001, 0.000	0.005	−0.001	−0.001, 0.000	0.003	−0.001	−0.001, 0.000	0.003	−0.001	−0.001, 0.000	0.002
**Height**	−0.001	−0.002, −0.001	0.001	−0.001	−0.002, −0.001	0.001	−0.001	−0.002, −0.001	0.001	−0.001	−0.002, −0.001	<0.001
**Adjusted R-squared %**	**16.91**			**16.74**			**17.84**			**17.92**		

Independent variables adjusted for based on p-value<0.20 are: gender, marital status, education, household income, height, asthma, heart attack, atrial fibrillation, coronary artery bypass graft, alcoholic drink days per month, number of general practice visits, urea, urate, cardiovascular medication use, anticholinergic medication use.

* β -coefficient;

† 95% confidence interval;

‡ p-value.

## Discussion

This population-based cross-sectional study of older Australian men and women examined the association of plasma L-arginine and the endogenous methylarginines, ADMA and SDMA with spirometric measures of lung function. In unadjusted analyses, ADMA and L-arginine/ADMA ratio were both statistically significantly associated with FEV1, FVC, and FEV1/FVC. These associations were attenuated but remained largely significant for ADMA and L-arginine/ADMA ratio with FEV1 and FVC with adjustment for a wide range of potential confounders. In none of the analyses was SDMA associated with any measures of spirometric lung function.

This research provides evidence to support the hypothesis that higher serum concentrations of the endogenous methylarginine ADMA are associated with lower measures of spirometric lung function in older adults. To the best of our knowledge, this is the first study to examine the association of serum L-arginine and the endogenous methylarginines with lung function in humans. The only known study to examine the effect of ADMA on airway hyperresponsiveness and lung function was performed in mice who received an osmotically delivered infusion of ADMA [22]. In this study ADMA infusion resulted in significantly enhanced lung resistance (RL) and decreased dynamic compliance (Cdyn) in response to methacholine. Furthermore, these physiologic changes were associated with significantly increased lung collagen content in the absence of inflammation. The results of this animal study support the findings from the current investigation and provide further evidence that higher concentrations of ADMA contribute to abnormal airway physiology.

Despite extensive data implicating NO in the maintenance of healthy airways and in the development of lung disease, there are very few data describing the effects of pharmacological NOS inhibitors on lung function in animals including humans. In one study, administration by aerosol of the NOS inhibitors, N-omega-nitro-L-arginine methyl ester (L-NAME) or NG-monomethyl-L-arginine (L-NMMA), to spontaneously breathing anesthetized guinea pigs resulted in a significant enhancement of lung resistance (RL) after increasing intravenous doses of histamine [32]. In another study L-NAME markedly augmented the response of artificially ventilated guinea pigs to aerosolized ovalbumin challenge, the potentiation of which could be prevented by NO in the inhaled air [33]. L-NAME has also been shown to inhibit the lipopolysaccharide induced airway hyporesponsiveness normally observed 9±12 h following bacterial exposure [34]. Moreover, L-NMMA attenuated both the ovalbumin-induced cellular influx and Evans blue leakage into the bronchoalveolar lavage (BAL) fluid and potentiated the hyperresponsiveness to methacholine in allergic Piebald-Virol-Glaxo rats [35]. More recently in a study involving asthmatic subjects, administration of nebulised L-NMMA resulted in a significant fall in peak exhaled NO compared with saline control values, which persisted for 4 hours. However, there were no effects of L-NMMA inhalation on heart rate, blood pressure, or FEV1 in either normal or asthmatic patients [36]. Wells SM et al used a mouse lung epithelial cell line to demonstrate that after LPS and cytokine stimulation, elevated ADMA inhibits NOS and contributes to the production of reactive oxygen and nitrogen species in vitro [22] that is characteristic of many lung diseases. In a randomised, double-blind, placebo-controlled, cross-over design study of 10 mildly asthmatic patients there was a statistically significant difference in the mean of the provocative dose producing a 20% fall in FEV1 to bradykinin between those receiving aerosol of L-NMMA or saline (placebo) [37]. L-NMMA also caused a decrease in the provocative concentration of methacholine producing a 20% fall in FEV1 from 0.93 mg/mL (range 0.12–2.55 mg/mL) to 0.38 mg/mL (range 0.06–0.92 mg/mL; p<0.01). The results suggest that bronchoconstriction after bradykinin inhalation is greatly inhibited by the formation of NO in airways of asthmatic patients and that NO could have a bronchoprotective role in asthma. As discussed previously, several animal studies have demonstrated that NOS inhibitors have provided support for iNOS-mediated NO production in the potentiation of airway inflammation [24]. In contrast, overexpression of eNOS attenuates airway inflammation in a murine model of allergic asthma [25] providing support for the hypothesis that eNOS-derived NO has a protective effect on airways while iNOS-induced NO augments airway inflammation. In the respiratory tract, NO is produced by a wide variety of cells, including epithelial cells, airway nerves, inflammatory cells, and vascular endothelial cells. Further studies will be necessary to identify the key cellular targets for NOS inhibition by ADMA as well as the relative involvement of each of the NOS isoforms.

The absence of an inverse association of SDMA with spirometric lung function measures in this study in multivariate analyses is surprising given that SDMA inhibits the cellular uptake of L-arginine, and could potentially exert its effects on lung function by depletion of the L-arginine pool which is required for adequate synthesis of NO. However, the absence of a significant inverse association between SDMA and lung function in the presence of a significant association with ADMA suggests that impairment in lung function is likely to be mediated through effects on NOS and not some other mechanism.

These findings are important given that other research that has demonstrated higher concentrations of serum ADMA are associated with OLD [27]. Reduced lung function is a risk factor for the development of OLD, hence confirmation that ADMA is associated with lung function impairment and/or decline in longitudinal studies might pave the way for interventional studies during this stage as a means of preventing decline. Given that ADMA concentrations can be modulated by non-pharmacological and pharmacological interventions it is important to determine if methylarginines are a potentially modifiable risk factor for impaired lung function and OLD.

This study has a number of strengths. The research was conducted in a relatively large population-based sample of community-dwelling older adults and hence the external validity of the findings is sound. Secondly, serum L-arginine, ADMA, and SDMA were all measured using hydrophilic-interaction liquid chromatography and isotope dilution tandem mass spectrometry, currently the gold standard for methylarginine measurement. The coefficients of variation of analytical runs were all within accepted limits and the hence the assays had good reliability. Finally, lung function was measured objectively with spirometry according to ATC guidelines [31].

Limitations of our study include its cross-sectional nature, which does not allow the assessment of cause-effect relationship between methylarginines and lung function. Hence, ADMA may simply be a marker of spirometric lung function. Moreover, similarly to most population studies on methylated arginines, the measurement of ADMA and SDMA from blood does not necessarily reflect intracellular concentrations of these compounds.

In summary, this study has shown that higher serum ADMA concentrations are associated with reduced spirometric measures of lung function while a higher L-arginine to ADMA ratio is associated with increased FEV1, increased percent of predicted FEV1, and increased percent of predicted FVC in an older population. Further research is needed to confirm if higher serum methylarginines are associated with the decline in lung function and incident obstructive lung disease.

## Supporting Information

Table S1 **Adjusted β -coefficient and 95% confidence intervals obtained from a multiple linear regression analysis of Asymmetric dimethylarginine, symmetric dimethylarginine, L-arginine, and L-arginine/asymmetric dimethylarginine ratio with percent predicted FEV1.**
(DOCX)

Table S2 **Adjusted β -coefficient and 95% confidence intervals obtained from a multiple linear regression analysis of Asymmetric dimethylarginine, symmetric dimethylarginine, L-arginine, and L-arginine/asymmetric dimethylarginine ratio with percent predicted FVC.**
(DOCX)
