# Supplementary material for: Serum Methylarginines and Spirometry-Measured Lung Function in Older Adults
Source: PLoS One. 2013 May 15;8(5):e58390. doi: 10.1371/journal.pone.0058390 (PMC3655195; doi:10.1371/journal.pone.0058390)
Supplement: Table S2 — Adjusted β -coefficient and 95% confidence intervals obtained from a multiple linear regression analysis of Asymmetric dimethylarginine, symmetric dimethylarginine, L-arginine, and L-arginine/asymmetric dimethylarginine ratio with percent predicted FVC. (DOCX) [file pone.0058390.s002.docx]

**Table S2**

| Characteristic | Adjusted | | | Adjusted | | | Adjusted | | | Adjusted | | |
| --- | --- | --- | --- | --- | --- | --- | --- | --- | --- | --- | --- | --- |
|  | β* | [95% CI]† | *p‡* | *β* | [95% CI] | *p* | *β* | [95% CI] | *p* | *β* | [95% CI] | *p* |
| Predictors | Asymmetric Dimethylarginine | | | Symmetric Dimethylarginine | | | L-Arginine | | | L-arginine/ADMA ratio | | |
| Q1 | Ref | | | Ref | | | Ref | | | Ref | | |
| Q2 | -2.89 | (-6.17, 0.399) | 0.085 | 1.503 | (-1.707, 4.713) | 0.358 | 0.885 | (-2.52, 4.29) | 0.61 | 1.508 | (-1.999, 5.016) | 0.398 |
| Q3 | -3.33 | (-6.7, 0.035) | 0.052 | 0.406 | (-3.202, 4.014) | 0.825 | 1.033 | (-2.344, 4.41) | 0.548 | 2.022 | (-1.355, 5.399) | 0.24 |
| Q4 | -2.5 | (-6.07, 1.078) | 0.171 | 0.307 | (-3.087, 3.7) | 0.859 | 2.962 | (-0.471, 6.396) | 0.091 | 4.051 | (0.659, 7.443) | 0.019 |
| Gender |  |  |  |  |  |  |  |  |  |  |  |  |
| Female | Ref | | | Ref | | | Ref | | | Ref | | |
| Male | -5.92 | (-8.53, -3.3) | <0.001 | -5.5 | (-8.171, -2.834) | <0.001 | -5.77 | (-8.339, -3.197) | <0.001 | -6.02 | (-8.615, -3.423) | <0.001 |
| Smoking years | -0.168 | (-0.264, -0.072) | 0.001 | -0.180 | (-0.277, -0.083) | <0.001 | -0.178 | (-0.274, -0.082) | <0.001 | -0.175 | (-0.271, -0.079) | <0.001 |
| Body mass index | -0.69 | (-0.95, -0.43) | <0.001 | -0.71 | (-0.982, -0.431) | <0.001 | -0.71 | (-0.972, -0.44) | <0.001 | -0.69 | (-0.953, -0.424) | <0.001 |
| Fibrinogen | -4.77 | (-7.13, -2.41) | <0.001 | -4.82 | (-7.165, -2.474) | <0.001 | -4.73 | (-7.052, -2.413) | <0.001 | -4.76 | (-7.057, -2.453) | <0.001 |
| Adjusted R-squared | 18.09 |  |  | 17.49 |  |  | 17.87 |  |  | 18.27 |  |  |

Independent variables adjusted for based on p-value < 0.20 are: cesd depressive symptoms score, gender, marital status, education, household income, body mass index, asthma, heart attack, atrial fibrillation, high cholesterol, hypertension, smoking years, alcoholic drink days per month, No. general practice visits, physical function score, urea, urate, white blood cell count, fasting glucose, fibrinogen, homocysteine, pulse pressure, C-reactive protein, cardiovascular medication use, anticholinergic medication use.

* β -coefficient; †95% confidence interval; ‡p-value
